# Geometric conservation laws for cells or vesicles with membrane nanotubes or singular points

**DOI:** 10.1186/1477-3155-4-6

**Published:** 2006-07-12

**Authors:** Yajun Yin, Jie Yin

**Affiliations:** 1 Department of Engineering Mechanics, School of Aerospace, FML, Tsinghua University, 100084, Beijing, China

## Abstract

On the basis of the integral theorems about the mean curvature and Gauss curvature, geometric conservation laws for cells or vesicles are proved. These conservation laws may depict various special bionano structures discovered in experiments, such as the membrane nanotubes and singular points grown from the surfaces of cells or vesicles. Potential applications of the conservation laws to lipid nanotube junctions that interconnect cells or vesicles are discussed.

## Background

Cell-to-cell communication is one of the focuses in cell biology. In the past, three mechanisms for intercellular communication, i.e. chemical synapses, gap junctions and plasmodesmata, have been confirmed. Recently, new mechanism for long-distance intercellular communication is revealed. Rustoms et al. [1] discover that highly sensitive nanotubular structures may be formed de novo between cells. Except for living cells, liposomes and lipid bilayer vesicles with membrane nanotubes have also been found in experiments [2-5]. Impressive photos of membrane nanotubes interconnecting vesicles can be seen in Ref.[3]. Another beautiful photo of a membrane nanotube generated from a vesicle deformed by optical tweezers can be shown in Ref.[4].

The above long-distance bionano structures may be of essential importance in cell biology and have drawn the attentions of researchers in different disciplines. Many annotations are concentrated on the formations of the membrane nanotubes. Different force generating processes such as the movement of motor proteins or the polymerization of cytoskeletal filaments have been suggested to be responsible for the tube formations in cells [6]. Of course, such annotations are absolutely necessary, but may not be sufficient. Another question with equal importance may be asked: Are there geometric conservation laws observed by such interesting bionano structures?

## Methods and results

To answer the above question, geometrical method will be used in this letter. As the first step, this paper will deal with the simplest "representative cell-nanotube element" (i.e. a cell or vesicle with membrane nanotubes). Then on the basis of the "element", vesicles with membrane nanotubes interconnected by a 2-way or 3-way nanotube junction will be investigated.

**Figure 1:**
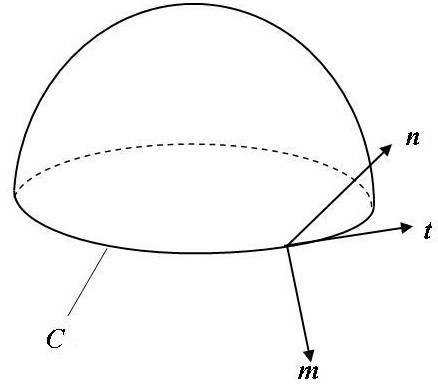
Schematic of the curved surface with unit vectors ***m***, ***t ***and ***n ***at its boundary.





Geometrically, a cell membrane or vesicle may be treated as a curved surface or 2D Riemann manifold. The generalized situation of a smooth curved surface is shown in Fig. 1. Let ***n ***be the outward unit normal of the surface and *C *be any smooth and closed curve drawn on the surface. On this curve, let ***m ***be the unit vector tangential to the surface and normal to the curve, drawn outward from the region enclosed by *C*. Let ***t ***be the unit tangent along the positive direction of the curve. Vectors ***t***, ***n ***and ***m ***form a right-handed system (Fig. 1) with the relation ***m = t ***× ***n ***satisfied. On such a surface, there are the conventional integral theorem about the mean curvature and a new integral theorem about the Gauss curvature [7]:

Here  and  are respectively the normal curvature and the geodesic torsion of curve *C*. *d****s ***= ***m****ds *is the vector element with *ds *the length element along the curve *C *. *H *= (*c*_1 _+ *c*_2_)/2 and *K *= *c*_1_*c*_2 _are respectively the mean curvature and Gauss curvature with *c*_1 _and *c*_2 _the two principle curvatures. *d****A ***= ***n****dA *is the element area vector in the normal direction of the curved surface and *A *is the area enclosed by *C*. For smooth and closed curved surfaces, Eq.(1) and Eq.(2) will degenerate respectively to  and . These integral theorems lay the foundation for the conservation law for cells or vesicles with membrane nanotubes or singular points.

**Figure 2:**
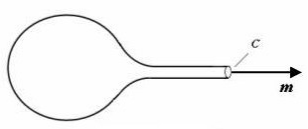
A cell or vesicle with one membrane nanotube.





Experiment [1] has shown that seamless transition is realized at the interconnecting location between cell membrane and membrane nanotube. From this information one may suppose that the cell and nanotube together has formed globally a smooth curved surface. If the tube is open and long enough, the open end of the curved surface may be idealized as part of a cylindrical surface with boundary curve *C *(Fig. 2). For simplicity *C *is supposed to be a plane curve perpendicular to the axis of the tube. Thus on curve *C τ*_*g *_= 0 will be met and the unit vector ***m ***may be parallel to the axis of the tube. At last the left-hand sides of Eq.(1) and Eq.(2) will become

Then Eq.(1) and Eq.(2) may be changed into





Here *r *is the radius of the tube. The unit vector ***m ***characterizes the "direction of the membrane nanotube". These are the geometric conservation laws for a cell or vesicle with one open membrane nanotube. Eq.(5) means that the integral of the mean curvature on the curved surface in Fig. 2 is dominated not only by the direction of the membrane nanotube but also by the radius of the tube. Eq.(6) shows that the integral of the Gauss curvature on the same curved surface is only determined by the direction of the membrane nanotube but independent of the radius of the tube. If the total number of membrane nanotubes on the cell or vesicle is *n*_*tube*_, then Eq.(5) and Eq.(6) may lead to





**Figure 3:**
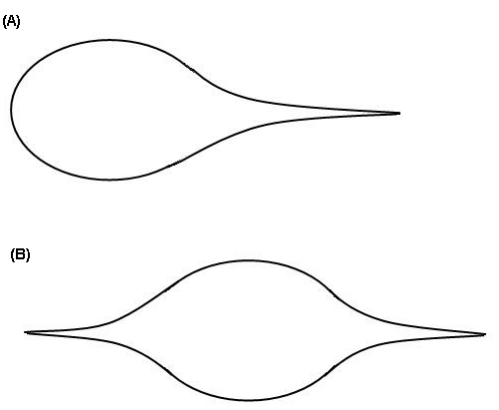
Cells or vesicles with one or two singular points.  (a) One singular point, (b) Two singular points.





Of course, in a living cell the membrane nanotube is seldom open and is usually closed at the tube's end point. Practical examples for such situation can be found in Refs.[1,4]. Geometrically this can be realized by letting the curve *C *converge gradually (i.e. *r *→ 0) and tangentially to a point at the tube axis. Hence the cell or vesicle with a closed membrane nanotube may be abstracted as a closed surface with a singular point (Fig. 3a). In practice, more than one singular point may exist on a cell or vesicle. A typical example for two singular points on a vesicle has been reported in Ref.[4] and may be schematically expressed in Fig. 3b. A cell with a group of singular points is displayed in Ref.[5]. If the total number of singular points on the cell or vesicle is *n*_*point*_, Eq.(7) and Eq.(8) may be rewritten as:

Here ***m***_*i *_is the direction of the *i*^th ^singular point. These are the geometric conservation laws for a cell or vesicle with singular points. Eq.(9) means that the integral of the mean curvature on the closed surface in Fig. 3 is always the vector zero. Eq.(10) implies that the integral of the Gauss curvature on the same surface is determined by the numbers and directions of singular points.

## Discussions

**Figure 4a:**
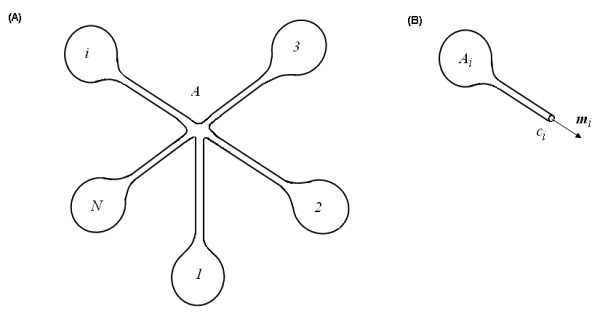
Smooth and closed curved surface *A*, abstracted from *N *vesicles with *N *nanotubes interconnected by a *N*-way nanotube junction. **(B)** The curved surface *A*_*i *_with a boundary *C*_*i*_, cut from the junction in Fig. 4a.





The above geometric conservation laws may be of potential applications to a kind of special bionano structures — lipid nanotube junctions. In recent years, the formation of vesicle-nanotube networks has become a focus [3,8]. In such networks, lipid nanotube junctions have been frequently used to interconnect vesicles and change network's topologies. However, our knowledge about this amazing bionano structure is still very limit. This limitation may be overcome in some extent with the aid of the geometric conservation laws. Here *N *vesicles with *N *lipid nanotubes interconnected at a junction will be studied (Fig. 4a). In this structure, every vesicle is supposed to have just one lipid nanotube and each vesicle-nanotube subsystem may be regarded as an open curved surface *A*_*i *_with a boundary *C*_*i *_(Fig. 4b). According to Eq.(5) and Eq.(6), one has

**Figure 5a:**
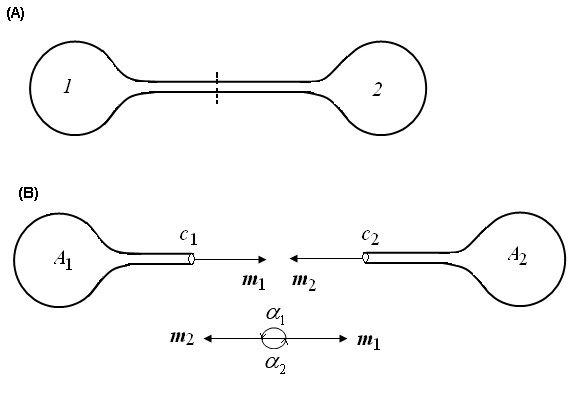
Smooth and closed curved surface abstracted from two vesicles with two nanotubes interconnected by a 2-way nanotube junction. **(B)** Two curved surfaces *A*_1 _and *A*_2 _with boundaries *C*_1 _and *C*_2_, cut from the junction in Fig. 5a.

Once *A*_*i *_are connected at *C*_*i *_(*i *= 1,2,......, *N*), the *N*-way nanotube junction may be generated through dynamic self-organizations. At equilibrium state, the vesicle-nanotube-junction system together may globally form a smooth and closed surface *A *on which the geometric conservation laws must be obeyed:





Eq.(13) and Eq.(14) are geometric regulations for the *N*-way nanotube junction. Here two special cases will be explored. The first case is *N *= 2 (Fig. 5a), which is correspondent to two vesicles *A*_1 _and *A*_2 _with lipid nanotubes connected at the tubes' ends *C*_1 _and *C*_2 _(Fig. 5b). Eq.(13) and Eq.(14) will lead to

*r*_1_***m***_1 _+ *r*_2_***m***_2 _= **0 **    (15)

***m***_1 _+ ***m***_2 _= **0 **    (16)

Eq.(15) and Eq.(16) may be equivalent to

*r*_1 _= *r*_2 _    (17)

*α*_1 _= *α*_2 _= 180°     (18)

Eq.(17) and Eq.(18) mean that the interconnecting section should be smooth and seamless. In another word, the axis of the nanotube should be a smooth curve. If this conclusion is combined with physical law, it may be further found that only straight nanotube instead of curved one is permissible, because the shortest distance between two points is the straight length and thus the straight nanotube may possess the lowest energy. In fact, all lipid nanotubes in experiments are straight without exceptions. This result may be used to direct micromanipulation. Practically, a lipid nanotube is drawn from one vesicle and then connected with another through various technologies such as micropipette-assisted technique and microelectrofusion method [8]. Theoretically, another possible micromanipulation process may exist: Two lipid nanotubes may be drawn simultaneously from two vesicles and then "welded" at the tubes' ends. In this case, Eq.(17) and Eq.(18) may tell us how to do successfully, i.e. not only the radii but also the axes of the two nanotubes should be kept consistent at the "welded" location.

**Figure 6a:**
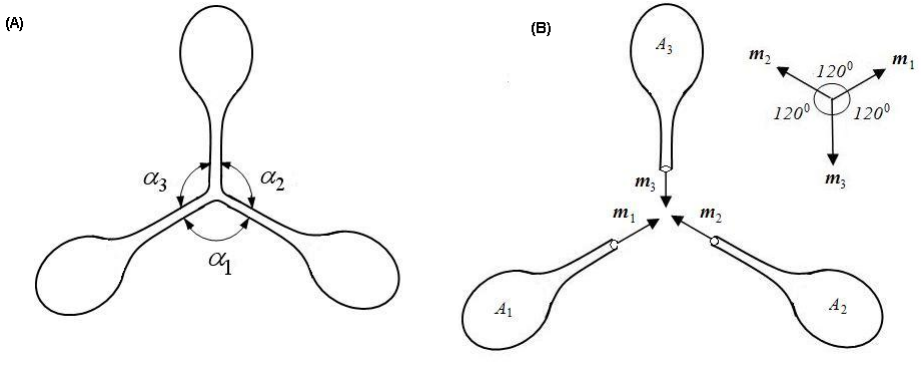
Smooth and closed curved surface formed from three vesicles with three nanotubes interconnected by a 3-way nanotube junction. **(B)** Three curved surfaces *A*_1_, *A*_2 _and *A*_3 _with boundaries *C*_1_, *C*_2 _and *C*_3_, cut from the junction in Fig. 6a.

The second case is *N *= 3 (Fig. 6a), which is correspondent to three vesicles *A*_1_, *A*_2 _and *A*_3 _with lipid nanotubes connected at the tubes' ends *C*_1_, *C*_2 _and *C*_3 _(Fig. 6b). In this case Eq.(13) and Eq.(14) will give

*r*_1_***m***_1 _+ *r*_2_***m***_2 _+ *r*_3_***m***_3 _= **0 **    (19)

***m***_1 _+ ***m***_2 _+ ***m***_3 _= **0 **    (20)

Eq.(19) and Eq.(20) will assure

*r*_1 _= *r*_2 _= *r*_3 _    (21)

*α*_1 _= *α*_2 _= *α*_3 _= 120°     (22)

Eq.(21) and Eq.(22) imply that the 3-way nanotube junction should be symmetric. Geometrically, the length of the nanotubes in the symmetric 3-way nanotube junction is the shortest among all possible 3-way junctions. Hence physically the symmetric one may be of the lowest energy. Fortunately, Eq.(21) and Eq.(22) coincides with experiments [3,8] very well.

In the cases of *N *≥ 4, the problems will become very complicated and will be explored in succeeding papers.

## Conclusion

In biology, many biostructures are constructed according to very simple geometrical regulations. This seems to be also true for cells or vesicles with membrane nanotubes or singular points. Once such laws are well understood, researchers in bionanotechnology field may benefit a lot from them.
